# Striving for meaning—Life in supported housing for people with psychiatric disabilities

**DOI:** 10.3402/qhw.v11.31249

**Published:** 2016-05-10

**Authors:** Rosita Brolin, David Brunt, Mikael Rask, Susanne Syrén, Anna Sandgren

**Affiliations:** 1 Department of Health and Caring Sciences, Linnaeus University, Växjö, Sweden; 2 The Center for Collaborative Palliative Care, Linnaeus University, Växjö, Sweden

**Keywords:** Grounded theory, psychiatric disabilities, self-determination, supported housing

## Abstract

The aim of this study was to develop a grounded theory about people with psychiatric disabilities, living in supported housing. Interviews as well as observations during the interviews were analyzed together with secondary analyses of quantitative and qualitative material from previous studies. *Being deprived of self-determination* emerged as the main concern for residents in supported housing and *striving for meaning* emerged as the pattern of behavior through which this group resolves their main concern. *Striving for meaning* involves living in the present, striving for self-determination, strengthening self-esteem, emotional processing and resting from the present. The strategies facilitate each other and are used singly, together simultaneously, or alternately. If they are successful, a meaning in the present is perceived. If all the strategies fail repeatedly, escaping from the present is used in order to deal with being deprived of self-determination. The implications of the findings suggest prioritizing a reduction of the institutional nature of the social and physical environment, and to support the residents’ self-determination.

Mental hospitals were the most common environment for the treatment of patients with psychiatric disabilities from the mid-1800s until the 1960s, and the duration of treatment was lifelong for many patients (Brunt, 2002). Psychiatric institutions were criticized for their uniformity, lack of freedom, and total control over the individual (Goffman, 1961). In Sweden, a Mental Health Reform was carried out in 1995, which followed a similar pattern that had been seen in many other Western countries, and which aimed to improve the life situation for people with long-term mental illness, now termed psychiatric disabilities. Long stays in psychiatric institutions were replaced with housing solutions in the community. In Sweden, two basic housing support models have been developed: housing support in the individual's own accommodation; and supported housing for those with a greater need for support (Brunt, 2002). In Sweden, approximately 26,000 people receive some form of housing support due to psychiatric disabilities (Swedish National Board of Health and Welfare, 2011), and about one-third live in a supported housing facility, entailing a congregate housing solution with varying in-house staff support. Most have their own private room or apartment but share common areas with other residents (Brunt, 2002). According to the Swedish National Board of Health and Welfare (2006), a psychiatric disability is a lasting psychiatric condition (>2 years) that involves not being able to manage everyday life on one's own. Psychosis, affective disorders, and neuropsychiatric disabilities are the most common diagnoses among those with a psychiatric disability in Sweden.

Supported housing has been shown to provide security and daily opportunities to socialize with other people (Brunt & Hansson 2002; Harvey, Killackey, Groves, & Herrman, 2012). On the other hand, the constant presence of fellow residents and staff can be perceived as an enforced togetherness, leading to an oppressed life and limited possibilities for integrity and privacy (Bengtsson-Tops, Ericsson, & Ehliasson, 2014; Harvey et al., 2012). Studies have shown that people with psychiatric disabilities prefer less restrictive housing solutions that give them opportunities for autonomy and freedom of choice in their everyday life (Nelson, Sylvestre, Aubry, George, & Trainor, 2007; Petersen, Hounsgaard, Borg, & Nielsen, 2012; Tsemberis, Rogers, Rodis, Dushuttle, & Skryha, 2003). In a questionnaire-based study in an earlier part of the current project, residents in supported housing in Sweden reported a moderate-to-high degree of satisfaction (Brolin, Rask, Syrén, Baigi, & Brunt, 2015), thus confirming the general picture seen in quantitative studies in the field (Elliott, Taylor, & Kearns, 1990; Hanrahan, Luchins, Savage, & Goldman, 2001; Tsemberis et al., 2003). However, this positive result needs to be considered in the light of the findings in a study, where previous negative experiences of psychiatric care settings could lead to low expectations for housing solutions, and thus to satisfaction with, and even expressions of gratitude for, poorer housing conditions that would not be acceptable to others in the community (Walker & Seasons, 2002).

The focus of the few studies that have been conducted in the research field has been on the residents’ perspectives on what they think about their housing situation. There is a considerable lack of explanatory studies of how the residents handle their situation in supported housing. The aim of the present study was therefore to develop a grounded theory of people with psychiatric disabilities, living in supported housing facilities. The research question guiding the study was thus: What is the main concern for people with psychiatric disabilities, living in supported housing, and how do they resolve this concern?

## Method

Classic grounded theory was chosen because of its inductiveness and explicit focus on the studied people's perspective, their main concern and how they handle their situation. The classic grounded theory, a general methodology (Glaser & Holton, 2004) in which both qualitative and quantitative data can be used (Glaser, 2001), does not adhere to any theoretical perspective (Glaser, 1998). It is an integrated assembly of hypotheses with the purpose of explaining and conceptualizing patterns of behavior (Glaser, 1978). In this study, it is thus the patterns of behavior of people with psychiatric disabilities living in supported housing that are explained. In accordance with the classic grounded theory (Glaser, 1998), no literature review was performed that focused on the main concern of the target group or how they handle their situation, prior to entering the research field. In order to raise the awareness of the researcher's preconceptions, the latter were written down prior to entering the research field.

The data collection was carried out in 2014 in an urban community (total population >130,000) and a rural community (total population <60,000) in Sweden. Participants were recruited from eight supported housing facilities. The social service managers recruited participants in five of these, while in three the first author was invited to inform all the residents about the study and recruit participants.

Interviews were conducted with 20 residents, living in supported housing facilities of various sizes, comprising 6–15 rooms or clustered apartments and some common areas, with in-house staff around the clock. The staffs, termed in this paper as care staff, are mainly nurses and assistant nurses. The participants that had lived in their current housing solution for 2–20 years were 21–66 years old and 11 were men. The interviews lasted 30–110 min and were conducted in the participants’ apartments, in a room in the housing facility, or outdoors. The first two interviews were tape-recorded, but in the following two interviews, the participants expressed that they did not want to participate if the interviews were to be tape-recorded. These interviews were thus performed with field notes only. Considering the vulnerability of the target group, a decision was made to perform the remaining interviews with field notes only, which were written during and immediately after the interviews in accordance with the methodological approach. Consequently, the following participants were not asked to approve tape-recording. Even though they had not received the question, several participants expressed that they did not want the interview to be tape-recorded.

A grounded theory is developed by focusing on repeated patterns in the data. Transcribing tape-recordings can generate a great many details that are not relevant for the development of a theory. The field notes included interviews and observations of, for example, environmental factors such as locked doors, residents being denied access to common areas, or staff wearing hospital clothes. The initial interview question was: *Would you like to tell me what it's like to live here?* which was followed-up by *What does that mean to you?* or *How do you feel about that?* in order to further encourage the residents to talk about what is important to them. Questions were also asked to the residents in order to validate the field notes: *I understand that you mean […] Have I got this correct?* The nature of the interviews was more of an open conversation than a formal interview. More detailed field notes and memos about ideas of concepts and possible interrelationships were written immediately after each interview, thus developing a rich memo bank. In the initial, open coding process, the field notes were analyzed line by line, and incidents in the data were identified, compared, and coded. Coding line by line facilitates avoiding hasty conclusions based on preconceptions or the preunderstanding of entire text passages. The constant comparative process while analyzing, served as guidance for the theoretical sampling on what data to collect and where to collect it, in order to saturate emerging concepts and the emerging theory (Glaser, 1978). For example, analyses of interviews in smaller facilities led to recruiting in larger ones and similarly with men leading to recruiting more women. New and more specific questions to ask in the following interviews emerged during the analysis, for example: *What makes you feel good?* and *What do you think about the future?* In order to maintain theoretical sensitivity and avoid description while collecting, coding, and analyzing data, a set of questions were asked to the data during the open-coding process: What is this data a study of? Is this an incident in the data or a preconceived understanding? Which category does this incident indicate? What is actually happening in the data? What is the residents’ main concern? How do they continually resolve this concern? The open codes were then compared to each other. Newly generated concepts were compared to new open codes and concepts were compared to other concepts in a constant comparative process in order to abstract the concepts and to identify their relationships with each other. The focus of this process was on finding a core concept that relates to as many of the other concepts as possible and thus explains, with as much variety as possible, how the main concern is continually resolved. When the core concept *striving for meaning* was found, the second phase, selective coding, began to delimit the coding to concepts related only to the core concept. Thus, the core concept was a template for further data collection and theoretical sampling (Glaser, 1978, 1998). In order to saturate the concepts, secondary analyzes were conducted on data that had been collected in earlier questionnaire based studies with preconceived questions (Brolin, Rask, Syrén, Baigi, & Brunt, 2015) and free-response questions (Brolin, Syrén, Rask, Sandgren, & Brunt, unpubl. data). These analyzes led to further clarifications of the concepts.

In the theoretical coding phase, more conceptual memos were written as theorizing ideas about the relationships between the concepts and the core concept. The memos were then sorted, memos on memos were written and sorted again and finally the theory *striving for meaning* was formulated. A literature review was then performed and used as a further source of data for saturation and clarification of the theory concepts (Glaser, 1998).

### Ethical considerations

The study was conducted in accordance with the Declaration of Helsinki (WMA, 2009) and was approved by the managers of social services in each municipality and by the Regional Ethical Review Board in Linköping (Reg. no. 2014/164-32). Prior to the interviews, the participants received both oral and written information about the study, including confidentiality, voluntary participation, being able to withdraw at any time and that non-participation or withdrawal would not affect their housing support or other services. The participants then gave written consent.

## Findings

*Being deprived of self-determination* emerged as the main concern of people with psychiatric disabilities who live in supported housing and *striving for meaning* emerged as the pattern of behavior through which they resolve their main concern. *Striving for meaning* is carried out by the strategies *living in the present*, *striving for self-determination*, *strengthening self-esteem*, *emotional processing*, and *resting from the present*. The strategies facilitate each other and are used singly, simultaneously together, or alternately. If they are successful, a meaning in the present is perceived. However, if all the strategies fail repeatedly, another strategy, *escaping from the present*, is used in order to deal with *being deprived of self-determination*.

*Being deprived of self-determination* means having limited options for influencing their own living situation. This can be due to: a housing form that provides limited privacy; sharing with people one has not chosen to; other people (e.g., care staff, physicians, administrators, and relatives) making decisions about one's current and future living situation, while being deprived of possibilities for influencing where to live; a physical environment that strengthens a feeling of being deprived of self-determination, for example, locked kitchens and TV rooms or staff wearing hospital clothing. Furthermore, the limited options for influencing the living situation may be affected by one's disabilities (difficulties concentrating, obsessions, phobias, etc.), and medication side-effects (fatigue, aggression, weight gain). *Being deprived of self-determination* leads to feelings of powerlessness, loss of meaning in life, and diminished self-esteem with feelings of being less worthy than other people in the community.

### Striving for meaning

*Being deprived of self-determination* is resolved by *striving for meaning*, which means searching for, or actively constructing meaning in the present, in a life situation that does not meet their actual needs, expectations, or desires. *Striving for meaning* is performed by *living in the present*, *striving for self-determination*, *strengthening self-esteem*, *emotional processing*, and/or *resting from the present*. It is important to emphasize that none of these strategies is better than any other and that all of them are valuable when striving for meaning. They facilitate each other and may be used singly, simultaneously together, or alternately. If they are successful, a meaning in the present is perceived. *Striving for meaning* can thus be seen as an ongoing process, where different strategies are used at the same time or one after the other. For example, when living in the present, striving for self-determination, strengthening the self-esteem, and/or emotional processing do not lead to meaning in the present, resting from the present is used to achieve a temporary break, to gain sufficient inspiration and strength to return to living in the present and the continuous striving for meaning. If all the aforementioned strategies used for striving for meaning repeatedly fail, *escaping from the present* may be the only way to manage the main concern, *being deprived of self-determination*. The strategies and their sub-strategies are presented in Table I.

**Table I:** The strategies and sub-strategies of the theory *Striving for meaning*.

Living in the present	Keeping busy Enjoying sensory perceptions
Striving for self-determination	Retaining self-determination Regaining self-determination Acquiring new forms of self-determination
Strengthening self-esteem	Seeking confirmation Allying Seeking affection
Emotional processing	Mastering thoughts Expressing emotions Confiding
Resting from the present	Resting in a fictional world Resting in the past Resting in the future
Escaping from the present	Living in a fictional world Living in the past Living in the future

### Living in the present

Living in the present means making the best of every moment here and now. Being deprived of self-determination, and therefore having limited options to change their lives, leads to limited possibilities for future planning. The residents realize that, even if they are not satisfied with their life situation, they have no other choice than to accept it, to make the best of it, and to take advantage of what is positive. Living in the present is performed by keeping busy and enjoying sensory perceptions.

*Keeping busy* means occupying themselves as much as possible through daily tasks such as housework and gardening, or leisure activities and hobbies. Living in the present through keeping busy was found to be easier in housing facilities located in rural areas compared to those in urban areas due to more space outside and more opportunities for activities such as gardening, carpentry, vehicle repairing, snow shoveling, etc. Being fully occupied helps pass the time, dispel thoughts, and find a meaning for the present.

*Enjoying sensory perceptions* means to enjoy visual and auditory impressions as well as sensations of taste and smell. The residents enjoy listening to music, the smell and taste of good food and good coffee or tea, smoking a cigarette, seeing the greenery, flowers and animals in nature. The pleasure of sensory perceptions provides meaning for the present and facilitates emotional processing.

Living in the present often contributes to feelings of meaning in the present. Keeping busy helps to strengthen one's self-esteem and may also provide new areas for self-determination, such as when, how and in what order to do housework, gardening or leisure activities. However, a lack of opportunities to occupy oneself during the daytime leads to reduced meaning in the present and increased feelings of deprived self-determination.

### Striving for self-determination

In order to make the present meaningful, opportunities for self-determination are sought. Striving for self-determination involves retaining, regaining, and acquiring new forms of self-determination.

*Retaining self-determination* means holding on to self-determination. This could, for example, be done by protecting one's rights to make decisions in one's own home, locking the door and not opening when someone is knocking, or forgoing certain kinds of help and support when there is no one who they trust.

*Regaining self-determination* means recapturing lost self-determination, which could for example be done by applying to move to another residence, requesting to discontinue medication and/or trying a different method of treatment.

*Acquiring new forms of self-determination* means finding new areas for self-determination, which could for example be done by deciding to become a vegetarian or supplementing an involuntary medical treatment with alternative therapies, such as herbal remedies and yoga.

The more self-determination conquered, the greater the potential for privacy and freedom, which in turn helps to strengthen self-esteem, living in the present and finding meaning in the present. However, if striving for self-determination fails, it leads to a weakened self-esteem with increased feelings of deprived self-determination and reduced meaning in the present.

### Strengthening self-esteem

While striving for meaning in the present, different strategies are used for strengthening self-esteem, which implies seeking confirmation, allying and seeking affection.

*Seeking confirmation* represents a desire to be important. Confirmation is sought by striving to be there for others and to help others, which in turn leads to feelings of being important. Confirmation is also sought by sharing experiences with others in conversations, letters, lectures or blogs, and by demonstrating skills and talents in art exhibitions, musical performances, or theater performances.

*Allying* entails seeking a connection, a togetherness or companionship with others. This is done in attempts to share interests and engage in activities with others, for example, shopping, cooking, television viewing, needle working, card games, playing music, or going to the theater. Togetherness can also be achieved in the context of common mealtimes, whether there are any conversations or not, during the meal.

*Seeking affection* means trying to find ways to satisfy the need for closeness and fondness. Affection is sought with relatives, but also with fellow residents. Keeping pets is another way to seek opportunities for affection.

Of these three strategies, seeking confirmation is the one that was found to be the most successful for strengthening self-esteem. To be recognized as talented or skilled strengthens self-esteem, which in turn facilitates living in the present and striving for self-determination. Confirmation from others also contributes to meaning but a belief in one's own talent or capability must already exist. Similarly, some belief in one's own ability to create and maintain contacts must already exist in order to use allying and seeking affection. It is otherwise impossible to use these strategies for strengthening self-esteem.

A strengthened self-esteem facilitates living in the present and contributes to meaning in the present. Failing in strengthening self-esteem, leads to reduced meaning in the present and increased feelings of deprived self-determination. Seeking affection seems to fail more often than allying, at least when seeking affection with other people. When affection is sought with a pet, the strategy more often increases the self-esteem, which in turn contributes to meaning in the present.

### Emotional processing

Emotional processing is an ongoing process that involves dealing with experiences of deprived self-determination such as powerlessness, loneliness, meaninglessness, to be misunderstood and unfairly treated, and anxiety about the future. Emotional processing also means dealing with experiences related to their own disabilities and medication side effects. Emotional processing involves mastering thoughts, expressing emotions, and confiding.

*Mastering thoughts* means gaining control over thoughts and feelings. This can be done by comparing the current living situation with previous negative experiences in order to replace negative thoughts about it with positive thoughts, thinking that this could have been worse. Mastering thoughts is also performed by waiting for the right opportunity to entrust ones thoughts until the “right person” is available, and by learning how to handle one's own disabilities, for example, phobias, anxiety, or obsessive actions, by challenging them.

*Expressing emotions* means venting experiences, thoughts, and feelings, and sharing them with others. This is done not only by, for example, laughing, crying, screaming, or smashing things but also by drawing or painting pictures; writing books, poems, diaries, blogs, or Web sites; composing their own pieces of music; playing instruments; or listening to music that reflects their mood.

*Confiding* involves sharing thoughts and feelings in face-to-face conversations with people they trust: staff, relatives, or fellow residents, but also with cats, dogs, and other pets who serve as reliable and good “listeners.”

When the strategies for emotional processing are successful, they facilitate living in the present, striving for self-determination and strengthening the self-esteem. However, if emotional processing fails, it leads to reduced meaning in the present and the feelings of being deprived of self-determination increase. Even if emotional processing fails, striving for meaning may continue through the other strategies: living in the present, striving for self-determination, or strengthening the self-esteem. However, if none of the strategies is successful feelings of frustration over being deprived of self-determination may be overwhelming. This is resolved through resting from the present to gather courage and strength sufficient to deal with the present again.

### Resting from the present

Resting from the present involves disconnecting all thoughts related to the present through resting in a fictional world, resting in the past, or resting in the future.

*Resting in a fictional world* means using various media, such as books, TV, movies, or video games, as ways of visiting a fictional world, which helps to pass time while providing access to a breathing space and a temporary rest from thoughts about the current situation, as well as a rest from worries about the future. The strategy also makes it easier for individuals to control themselves and not show others how they feel.

*Resting in the past* involves daydreaming about previous positive experiences, such as former good relations, positive housing situations, or day trips and vacation trips. Daydreaming about the good times in the past provides a breathing space and a temporary rest from the present as well as from worries for the future.

*Resting in the future* involves daydreams about a better life in the future. These may involve a future companionship or profession, as well as a future home or the ability to have pets. The positive daydreams about the future also provide a breathing space and a temporary rest from the present and from worries for the future.

Courage and strength can be retrieved through the temporary rest, which in turn facilitates returning to the present and the continuous striving for meaning. The fictitious reality, as well as thoughts on previous positive experiences can inspire dreams and fantasies about the future. These in turn can act as triggers for daring to seek new ways for self-determination to achieve the dreams. However, leaving the fictional world or daydreams and returning to the present may be stressful while being deprived of self-determination. Then the strategies for emotional processing appear to be needed, in order to facilitate the return to the present and the continuous striving for meaning.

### Escaping from the present

Escaping from the present means remaining in the rest from the present. If the strategies, living in the present, striving for self-determination, strengthening self-esteem and emotional processing, fail repeatedly, the feelings of being deprived of self-determination increase. This in turn leads to resignation and, if no ways for finding meaning in the present are visible, escaping from the present may be the only way to deal with being deprived of self-determination. This is done through the same strategies as used in resting from the present, but is not just a temporary rest, they remain in these strategies with very few short returns to the present. Escaping from the present is therefore done through *living in a fictional world*, *living in the past*, or *living in the future*. It means spending whole days in a fictional world or in daydreams about the past or the future, in order to survive when the present constitutes a constant reminder of being deprived of self-determination. Escaping from the present involves a life pattern alternating between watching TV or movies, playing video games, reading books, daydreaming, eating, and sleeping. The meals are the only interruptions from the fictional world or daydreams. During meals, the present is visited for a short moment of pleasure, consisting of the taste perception, immediately followed by an escape back to the fictional world or daydreams. Apart from the meals, the meaning in life is then represented by what is happening in the fictional world or in daydreams.

## Discussion

In this grounded theory, *Striving for meaning* emerged as the pattern of behavior through which people with psychiatric disabilities living in supported housing facilities resolve their main concern, *being deprived of self-determination*. It should be emphasized that striving for meaning does not represent all the patterns of behavior of the residents in supported housing but is one important pattern of behavior in which they are engaged.

The authors’ prior understanding should be discussed as the authors have performed previous studies in the context of supported housing for people with psychiatric disabilities. However, those studies had their focus on the psychosocial environment (Brunt, 2002; Brunt & Hansson, 2002) and on satisfaction with housing situation (Brolin, Rask, Syrén, Baigi, & Brunt, 2015), while the focus of the current study is on the main concern for the residents. No literature review was performed about the main concern for people with psychiatric disabilities and/or how they handle their situation, but notes about the researcher's preconceptions were written down prior to entering the research field.

A classic grounded theory should be judged by fit, relevance, workability, and modifiability (Glaser, 1978, 1998). Fit means how closely the concepts of the theory fit with the incidents that they are representing. In order to achieve this, conceptual memos were written and sorted throughout the analysis process, seeking relationships between concepts and incidents as well as between concepts and the core concept. The interviews were not audio-taped, which could be discussed because field notes do not include every detail in the interviews. However, in the classic grounded theory, the researcher is not looking for details but for common concerns and repeated patterns in data, and recorded and transcribed interviews may increase the risk that the researcher focuses more on individual details than on repeating patterns in data (Glaser, 1978, 1998). Furthermore, the target group is a vulnerable one and several participants expressed that they did not want the interview to be tape-recorded and some even stated explicitly that no tape-recording was a prerequisite for participating. In order to achieve relevance, that is, the real concern of the participants, data was collected and coded until nothing new emerged. One limitation of this study is the role of the social service managers, who by facilitating contact with the participants could be seen as gatekeepers potentially hindering some residents from participating in the study. There were, however, no other ways to gain access to the participants, except in three facilities, where all the residents were invited to receive information about the study. Workability means that the theory explains how the concern is resolved. The focus of the analysis was thus on finding a core concept that explains with as much variation as possible how the main concern is resolved. A grounded theory is abstract of time, place, and people, and can be modified. The proposed theory is developed from data collected in the context of supported housing facilities for people with psychiatric disabilities. The theory explains common human behavior, however, and might be relevant in other contexts and can contribute to understanding how people strive for meaning when deprived of self-determination. Further research is, however, needed to determine whether the theory fits other substantive areas, where new concepts could emerge to modify the theory *striving for meaning*, to optimize the fit.

The main concern, *being deprived of self-determination*, is reminiscent of results from Goffman's (1961) studies of life in institutions such as prisons, army camps, and mental hospitals. Goffman found that all aspects of individuals’ lives were subordinate to and dependent on the institution's organization and authority. Similar results can be found in more recent research from psychiatric settings, for example, in terms of: “Leading an oppressed life” in supported housing (Bengtsson-Tops et al., 2014), and “being between empowerment and powerlessness” in psychiatric inpatient care (Tveiten, Haukland, & Onstad, 2011). This has also been seen concerning other groups in society with, for example, experiences of powerlessness among the elderly in nursing homes (Parnell, 2005) and disempowerment among young people with disabilities in foster care (Geenen, Powers, Hogansen, & Pittman, 2007).

The Swedish Mental Health Reform was carried out in 1995 with the aim of improving the situation for people with psychiatric disabilities by replacing inpatient care in mental hospitals with housing solutions in the community. The setting and external physical attributes have thus changed but 20 years on the internal attributes, in terms of deprived self-determination, appear not to be dissimilar to those described by Goffman (1961) more than 50 years ago. The participants’ main concern can also be reflected in studies of housing preferences among people with psychiatric disabilities, who preferred less restrictive housing solutions that provided them with opportunities for autonomy and freedom of choice in their everyday life (Nelson et al., 2007; Petersen et al., 2012; Tsemberis et al., 2003). This study thus indicates that self-determination is still restricted in congregate psychiatric settings. The housing context, as in this study, constitutes an arena where the residents with psychiatric disabilities should be able to experience self-determination if the aims of the Swedish Mental Health Reform and support services are to be fulfilled. In order to reduce feelings of deprived self-determination in congregate-supported housing facilities, care staff should first identify areas in everyday life where restrictions occur that can prevent self-determination and then work to remove them or replace them with alternatives that can stimulate independence. Visible attributes, such as those mentioned above, hospital clothing, and locked rooms, are examples of the type of areas where changes can be made.

The concepts in this grounded theory *Striving for meaning* are by no means new. Meaning is an established feature in understanding human existence. It is a core concept in phenomenological philosophy (Husserl, 1970/1900; Merleau-Ponty, 1995). The meaning of human existence is in focus in existentialism and existential therapy, Frankl (2006/1947) for instance, argued that the quest for meaning is the primary, most powerful, motivating and driving force in human lives. Furthermore Goffman (1961) described how mental patients and prisoners in the 1960s used different coping strategies, comparable to the concepts in this grounded theory in order to live and survive in the closed worlds of the institutions. For example, they engaged in different kinds of chores which could be compared to keeping busy in this grounded theory, and enjoyed the few satisfactions that were available, which is similar to enjoying sensory perceptions. Furthermore, they formed pairs or groups with strong mutual ties, which is reminiscent to allying in this grounded theory, they refused to cooperate with the staff, which could be seen as a way of striving for self-determination and they used TV or books as a form of escapism, comparable to resting in the present or escaping from the present. The concepts in the theory *Striving for meaning* can also be compared to those reported in modern psychiatric literature. Living in the present by keeping busy is comparable to “having a meaningful daily occupation” (Goldberg, Brintell, & Goldberg, 2002) or finding meaning in everyday activities (Argentzell, Håkansson, & Eklund, 2012). Striving for self-determination has similarities to “a struggle for self-determination” in a mental health rehabilitation context (Petersen et al., 2012), while strengthening self-esteem is comparable to “feeling competent and accepted by society” (Argentzell et al., 2012).

Through its explanatory structure, the theory striving for meaning can contribute to greater understanding for the residents’ situation and patterns of behavior. The theory may serve as a tool for the residents in supported housing to understand their own situation, and at the same time for care staff in their work to reduce the residents’ feelings of being deprived of self-determination and to facilitate their strategies in striving for meaning. For example, the care staff can facilitate living in the present by being responsive to each individual's unique preferences for meaningful activities and to create opportunities for these. The care staff can strengthen self-esteem by creating opportunities for the residents to demonstrate their results of these activities. Furthermore, the care staff can encourage and support the residents in making their own decisions in everyday life and also encourage and support them to seek necessary information for this task. In addition, a caring relationship based on trust and respect facilitates emotional processing. Resting from the present represents a strategy to achieve a necessary temporary break from feelings of being deprived of self-determination, while escaping from the present represents a strategy to survive, by spending whole days in a fictional world or in daydreams. The concepts resting from the present and escaping from the present can be compared to “escapism,” a pattern of behavior among online gamers to avoid everyday hassles and psychological distress (Hagström & Kaldo, 2014; Kardefelt-Winther, 2014). The care staff may thus face a dilemma where they, on the one hand, encourage residents to make their own decisions in striving for meaning, while on the other have to be aware of the potential health risks, both mentally (Hagström & Kaldo, 2014) and physically (Lee et al., 2012), of spending days in bed, in front of the TV or in video games, and should perhaps suggest alternative less potentially negative strategies.

## Conclusion

The theory *Striving for meaning* can be understood as an ongoing process in which the strategies, living in the present, striving for self-determination, strengthening self-esteem, emotional processing, resting from the present, and/or escaping from the present, are used in order to handle the main concern, being deprived of self-determination in a life situation that does not meet needs, expectations or desires.

The main concern of people with psychiatric disabilities living in supported housing facilities, being deprived of self-determination, is a serious critique of those housing services for the target group. The study reveals that similar experiences have been a feature of psychiatric settings for a very long time and still need to be attended to in order to achieve the goals of political reforms. The implications of the results suggest that measures need to be taken on two levels: first, politicians and administrators need to create the attributes and structures that support rather than restrict self-determination by reducing the institutional nature of the environment. Second, care staff need to identify the residents’ strategies for trying to resolve their main concern in everyday life, as exemplified in this study, and also identify the reasons for these strategies and to support the residents in achieving their aim. Further research is needed, perhaps in the form of observational studies, in order to gain greater knowledge of the residents’ strategies and the staff's responses. The theory *Striving for meaning* can be used as a base for future intervention studies in the field.
